# Evaluation of the molluscicidal activities of arylpyrrole on *Oncomelania hupensis*, the intermediate host of *Schistosoma japonicum*

**DOI:** 10.7717/peerj.12209

**Published:** 2021-09-27

**Authors:** Yuntian Xing, Jiakai Yao, Guoli Qu, Jianrong Dai, Bainian Feng

**Affiliations:** 1 School of Pharmaceutical Sciences, Jiangnan University, Wuxi, China; 2 National Health Commission Key Laboratory of Parasitic Disease Control and Prevention, Jiangsu Provincial Key Laboratory on Parasite and Vector Control Technology, Jiangsu Institute of Parasitic Diseases, Wuxi, China

**Keywords:** Arylpyrrole, *Oncomelania hupensis*, *Schistosoma japonicum*, Molluscicide

## Abstract

The snail *Oncomelania hupensis* is the only intermediate host of the highly invasive parasite *Schistosoma japonicum*. Molluscicide is often used to curb transmission of *S. japonicum*. Niclosamide, the only World Health Organization (WHO) recognized molluscicide, presents major drawbacks, including high cost and toxicity towards aquatic animals. In the present study, a number of aryl pyrrole derivatives (ADs) were synthesized to serve as potential molluscicides and were tested on *O. hupensis*. To uncover the underlying mechanisms, adenosine triphosphate (ATP) and adenosine diphosphate (ADP) levels were assessed in the soft body of ADs-exposed *O. hupensis*, using high performance liquid chromatography (HPLC). The effect of C6 on key points of energy metabolism (the activities of complexes I, III, IV and the membrane potential) was determined. We demonstrated that the Compound 6 (C6, 4-bromo-1-(bromomethyl)-2-(4-chlorophenyl)-5-(trifluoromethyl)-1*H*-pyrrole-3-carbonitrile) exerted the strongest molluscicidal activity against adult *O. hupensis* at LC_50_ of 0.27, 0.19, and 0.13 mg/L for 24, 48, and 72 h respectively. Moreover, we found that the bromide on the pyrrole ring of C6 was essential for molluscicidal activity. Furthermore, the ATP content reduced from 194.46 to 139.75 μg/g after exposure to 1/2 LC_50_, and reduced to 93.06 μg/g after exposure to LC_50_. ADP, on the other hand, remained the same level before and after C6 exposure. We found that C6, at 1/2 LC_50,_ reduced the membrane potential of *O. hupensis*, while no significant changes were observed in the activities of complexes I, III, and IV. C6 was identified with excellent activities on *O. hupensis*. The obtained structure−activity relationship and action mechanism study results should be useful for further compound design and development.

## Introduction

*Schistosomiasis* is a highly prevalent tropical parasitic disease, with a global incidence of 250 million (Barsoum, Esmat & El-Baz, 2013). The most common schistosomiasis species in China is *Schistosoma japonicum* and its only intermediary host is the snail *O. hupensis* (Shan et al., 2020; Sun et al., 2020). One proposed approach of managing this parasite is to eliminate the intermediary host, *i.e., O. hupensis* (Yang et al., 2008; Zhang & Zou, 2020). Niclosamide is a commonly used synthetic molluscicides in China (Dai et al., 2014; Dai et al., 2015; Yang et al., 2019). However, Niclosamide has been used to control the *O. hupensis* population in China for over 30 years. The annual usage of Niclosamide is about 3,200 ton, nationwide. The long-term and large-scale use of Niclosamide can increase the risk of drug resistance. One proposed approach of reducing the risk of drug resistance is drug rotation strategy (Rigby et al., 2021). However, the proved molluscicides are only Niclosamide, metaldehyde and Luo-Wei (Jia et al., 2019). There is an urgent need to establish new molluscicides that may support molluscicide rotation strategy. Emerging evidences revealed that the use of natural compounds as novel molluscicides may be the best and most effective option (El-Emam et al., 2020). Aryl pyrrole is a pharmacophoric structure commonly presenting with insecticidal, acaricidal, and fungicidal properties (Wu et al., 2020). Aryl pyrrole was first synthesized from dioxapyrrolomycin, an essential compound in *Streptomyces* metabolism, in 1987 (Carter et al., 1987). However, its molluscicidal activity, if any, has not been evaluated. Herein, we designed and synthesized a number of aryl pyrrole derivatives (ADs), their molluscicidal activity against *O. hupensis*. The possible action mechanism, illustrated by change of the ATP production by *O. hupensis*, was also reported.

## Materials & Methods

### Chemistry

**Figure 1:**
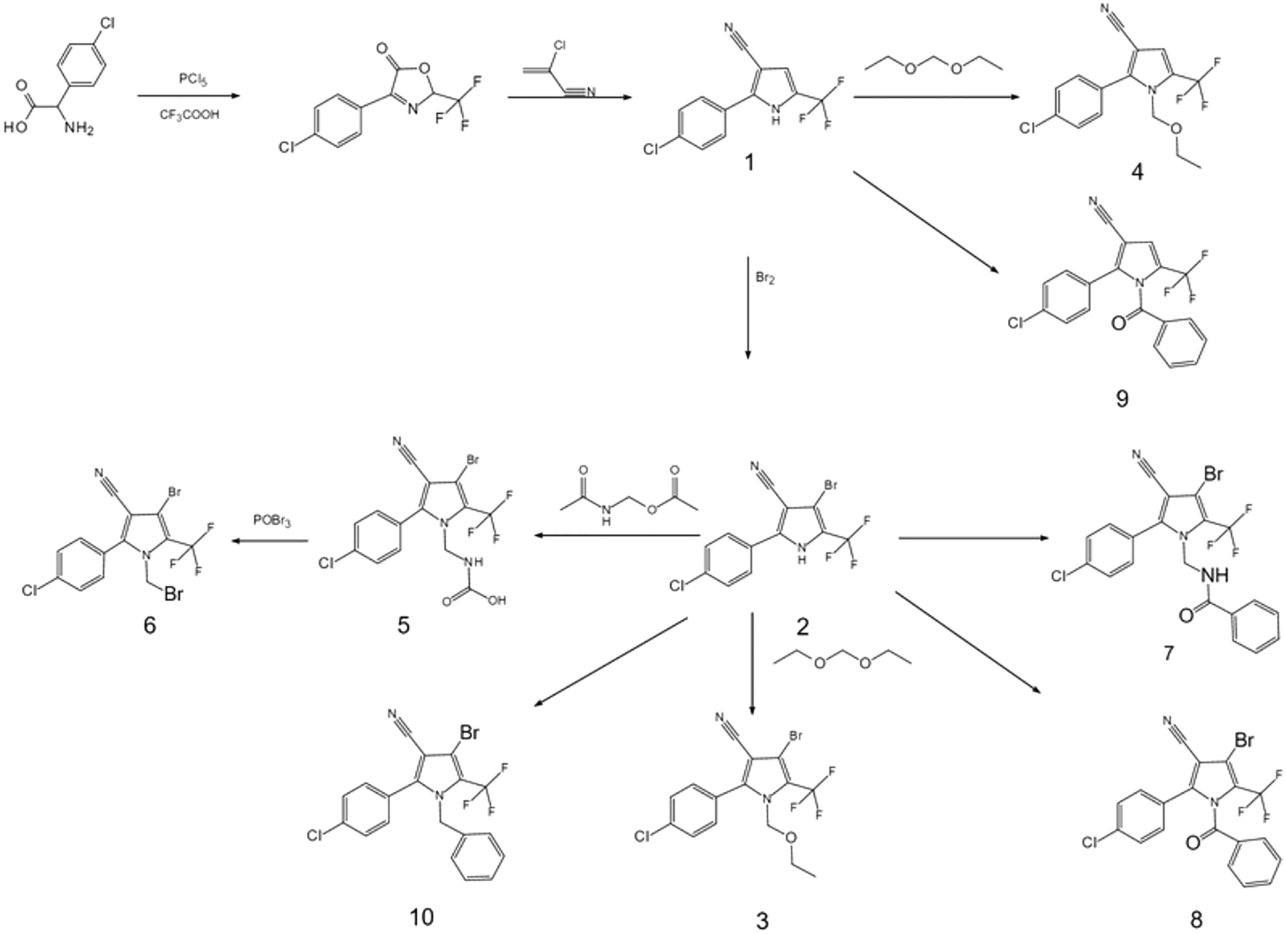
The synthetic route of arylpyrrole.

The procedure of aryl pyrrole synthesis is summarized in Fig. 1.

### 2-(4-chlorophenyl)-5-(trifluoromethyl)-1 *H*-pyrrole-3-carbonitrile (Compound 1, C1)

2-amino-2-(4-chlorophenyl) acetic acid (13.06 g) was dissolved in 30 mL acetonitrile with trifluoroacetic acid (2.6 g) and triethylamine (1.7 g) and stirred continuously for 15 min. Subsequently, PCl5 (3.6 g) was introduced and the solution was further stirred at 40 °C for 1 h and at 60 °C for 4 h. Next, the solvent was removed, using reduced pressure, followed by the addition of toluene (15 mL), and washing by 15 mL NaHCO_3_ (15 mL) and water (15 mL). The organic phase was dried using anhydrous sodium sulfate. Any remaining solvent was removed *via* evaporation under reduced pressure at 60 °C and the desired product (3.6 g) was acquired. It was then dissolved in acetonitrile (13 mL) and 2-chloroacrylonitrile (1.42 g) was added and stirred. Then, the mixture of triethylamine (1.63 g) and acetonitrile (3 mL) were introduced drop by drop and the solution was heated to 45 °C for 1 h before cooling to room temperature (RT). The solvent was removed using reduced pressure and water (20 mL) was added to halt the reaction. The crude product was acquired by filtering, washing with water (10 mL) 3X, and drying at 80 °C. The product yield was 85%. The product identification was done as follows: FT-IR (cm^−1^): 3168, 2237, 1471, 1425, 1339, 1307, 1202, 1170, 1109, 1097, 1013, 953, 827, 732, 651, 625; ^1^H NMR (400 MHz, CDCl_3_) δ 9.10 (s, 1H, NH), 7.69 (d, *J* = 8.6 Hz, 2H, ArH), 7.53 (d, *J* = 8.6 Hz, 2H, ArH), 6.96 (s, 1H, ArH).

### 4-bromo-2-(4-chlorophenyl)-5-(trifluoromethyl)-1 *H*-pyrrole-3-carbonitrile (Compound 2, C2)

The previous product (3.5 g) was re-suspended in chloroform (30 mL) and kept at <40 °C, while bromine (5.4 g) was slowly dripped into the solution. After 0.5 h, the resulting solution was heated to 30 °C and maintained for 4 h. Next, solvent was removed under reduced pressure at 60 °C. The crude product was acquired by filtering, washing with water (10 mL) 3X, and drying at 80 °C. The product yield was 93%. The product identification was done as follows: FT-IR (cm^1^): 3149, 2234, 1467, 1418, 1333, 1309, 1171, 1107, 1011, 975, 825, 734, 652; ^1^H NMR (400 MHz, CDCl_3_) δ 9.21 (s, 1H, NH), 7.65 (d, *J* = 8.6 Hz, 2H, ArH), 7.51 (d, *J* = 8.6 Hz, 2H, ArH).

### 4-bromo-2-(4-chlorophenyl)-1-(ethoxymethyl)-5-(trifluoromethyl)-1 *H*-pyrrole-3-carbonitrile (Compound 3, C3)

The previous product (4.2 g) was re-suspended in toluene (12 mL) and DMF (1.2 mL) and maintained at <40 °C. Next, POCl_3_ (2.25 g) was dripped slowly into the solution before heating at 50 °C for 30 min. Triethylamine (1.5 g) was dripped slowly when the temperature dropped to 30 °C. Next, water (20 mL) was added to the solution and it was heated back up to 40 °C and maintained for an hour, followed by removal of the solvent *via* evaporation under reduced pressure at 60 °C. The crude product was acquired by filtering, washing with water (10 mL) 3X, and drying at 80 °C. The product yield was 90%. The product identification was done as follows: FT-IR (cm^−1^): 1349, 1206, 1170, 1122, 1094, 1054, 832, 730; ^1^H NMR (400 MHz, CDCl_3_) δ 7.53 (d, *J* = 8.6 Hz, 2H, ArH), 7.48 (d, *J* = 8.7 Hz, 2H, ArH), 5.20 (s, 2H, CH_2_), 3.40 (q, *J* = 7.0 Hz, 2H, CH_2_), 1.17 (t, *J* = 7.0 Hz, 3H, CH_3_).

### 2-(4-chlorophenyl)-1-(ethoxymethyl)-5-(trifluoromethyl)-1 *H*-pyrrole-3-carbonitrile (Compound 4, C4)

C1 (4.3 g) was treated in the same procedure as described for synthesis of C3. The product yield was 90%. The product identification was done as follows: FT-IR (cm^−1^): 2230, 1576, 1474, 1417, 1351, 1270, 1207, 1175, 1121, 1091, 1047, 900, 839, 726, 678; ^1^H NMR (400 MHz, CDCl_3_) δ 7.52 (s, 4H, ArH), 6.96 (s, 1H, ArH), 5.19 (s, 2H, CH_2_), 3.44 (q, *J* = 7.0 Hz, 2H, CH_2_), 1.18 (t, *J* = 7.0 Hz, 3H, CH_3_).

### *N*-((3-bromo-5-(4-chlorophenyl)-4-cyano-2-(trifluoromethyl)-1 *H*-pyrrol-1-yl) methyl) acetamide (Compound 5, C5)

(4-bromo-2-(4-chlorophenyl)-5-(trifluoromethyl)-1H-pyrrole-3-carbonitrile, 1.75 g) was dissolved in THF (6 mL) and stirred 10 °C for 20 min. Next, NaH (activated, 0.44 g) was added. Solution A was obtained after stirring for 15 min. Next, the substrate methyl acetoacetate (0.78 g) was dissolved in THF (4 mL) and solution A was dripped slowly with stirring at 50 °C, followed by incubation at 70 °C for 4 h with reflux condensation. The product was extracted with water (5 mL) and ethyl acetate (8 mL) 3X. In addition, the ethyl acetate was collected and dried with anhydrous sodium sulfate. The crude product was acquired after evaporating the solvent under reduced pressure at 60 °C. The product yield was 40%. The product identification was done as follows: FT-IR (cm^−1^): 3255, 2232, 1653, 1550, 1481, 1430, 1400, 1354, 1263, 1195, 1171, 1114, 1094, 1057, 843, 827, 730, 682, 598; ^1^H NMR (400 MHz, CDCl_3_) δ 7.53 (d, *J* = 8.5 Hz, 2H, ArH), 7.41 (d, *J* = 8.5 Hz, 2H, ArH), 6.24 (s, 1H, NH), 5.37 (d, *J* = 6.4 Hz, 2H, CH_2_), 1.92 (s, 3H, CH_3_).

### 4-bromo-1-(bromomethyl)-2-(4-chlorophenyl)-5-(trifluoromethyl)-1 *H*-pyrrole-3-carbonitrile (Compound 6, C6)

The previous product (1.22 g) and phosphorus tribromoxide (2.35 g) were re-suspended in acetonitrile (5 mL) and maintained at 80 °C for 1 h. Next, acetonitrile was evaporated under reduced pressure at 60 °C and the product was extracted with water (5 mL) and ethyl acetate (8 mL) 3X. In addition, ethyl acetate was collected and evaporated under reduced pressure. The product was acquired using column chromatography. The product yield was 80%. The product identification was done as follows: FT-IR (cm^−1^): 2233, 1555, 1481, 1452, 1423, 1396, 1346, 1275, 1239, 1175, 1121, 1094, 1061, 854, 726, 621; 1H NMR (400 MHz, CDCl_3_) δ 7.57 (q, *J* = 8.5 Hz, 4H, ArH), 5.61 (s, 2H, CH_2_).

### *N*-((3-bromo-5(4-chlorophenyl)-4-cyano-2-(trifluoromethyl)-1 *H*-pyrrol-1-yl)methyl)benzamide (Compound 7, C7)

C2 (0.175 g) was re-suspended in DMF (dried, 1 mL). Next, NaH (0.66 g) was introduced and the resulting solution stirred for 20 min under the protection of nitrogen. Benzamidomethyl acetate (0.116 g) was added to the mixture. Next, the solution was maintained at 80 °C for 4 h and water was added to stop the reaction. The product was extracted using ethyl acetate and column chromatography. The product yield was 88%. The product identification was done as follows: FT-IR (cm^−1^): 1647, 1533, 1476, 1403, 1340, 1283, 1242, 1202, 1157, 1127, 1110, 1057, 831, 698; ^1^H NMR (400 MHz, CDCl_3_) δ 7.65 (d, *J* = 7.8 Hz, 2H, ArH), 7.55 (d, *J* = 8.3 Hz, 3H, ArH), 7.48–7.41 (m, 4H, ArH), 6.86 (s, 1H, NH), 5.60 (d, *J* = 6.3 Hz, 2H, CH_2_).

### 1-benzoyl-4-bromo-2-(4-chlorophenyl)-5-(trifluoromethyl)-1 *H*-pyrrole-3-carbonitrile (Compound 8, C8)

C2 (0.175g) and benzoyl chloride (0.141 g) were used to synthesize C8. The reaction was carried out in the same way as described for the synthesis of C7. The product yield was 91%. The product identification was done as follows: FT-IR (cm^−1^): 2825, 1785, 1682, 1599, 1453, 1419, 1323, 1287, 1210, 1173, 1127, 1015, 933, 804, 702, 666, 546; ^1^H NMR (400 MHz, CDCl_3_) δ 8.17 (d, *J* = 7.2 Hz, 2H, ArH), 7.69 (t, *J* = 7.4 Hz, 1H, ArH), 7.54 (t, *J* = 8.0 Hz, 3H, ArH), 7.40 (t, *J* = 7.8 Hz, 1H, ArH), 7.29 (s, 2H, ArH).

### 1-benzoyl-2-(4-chlorophenyl)-5-(trifluoromethyl)-1 *H*-pyrrole-3-carbonitrile (Compound 9, C9)

C1 (0.175 g) and benzoyl chloride (0.141 g) were used. The reaction was carried out in the same way as described for the synthesis of C7. The product yield was 90%. The product identification was done as follows: FT-IR (cm^−1^): 1784, 1683, 1599, 1452, 1419, 1277, 1210, 1173, 1126, 995, 803, 701, 666, 544; ^1^H NMR (400 MHz, DMSO) δ 8.15 (s, 2H, ArH), 7.95 (d, *J* = 7.6 Hz, 1H, ArH), 7.82 (t, *J* = 7.4 Hz, 2H, ArH), 7.65 (t, *J* = 7.8 Hz, 4H, ArH), 7.50 (t, *J* = 7.4 Hz, 1H, ArH).

### 1-benzyl-4-bromo-2-(4-chlorophenyl)-5-(trifluoromethyl)-1 *H*-pyrrole-3-carbonitrile (Compound 10, C10)

C2 (0.175 g) and benzyl bromide (0.171 g) were used to synthesize the C10. The reaction was carried out in the same way as described for the synthesis of C7. The product yield was 92%. The product identification was done as follows: FT-IR (cm^−1^): 2231, 1544, 1453, 1430, 1396, 1352, 1279, 1167, 1122, 1057, 1031, 830, 741, 726, 696; ^1^H NMR (400 MHz, CDCl_3_) δ 7.39 (d, *J* = 8.3 Hz, 2H, ArH), 7.29 (d, *J* = 6.5 Hz, 3H, ArH), 7.21 (d, *J* = 8.3 Hz, 2H, ArH), 6.79 (d, *J* = 5.4 Hz, 2H, ArH), 5.23 (s, 2H, CH_2_).

### Snails

Adult *O. hupensis* snails were gathered from the rural marshland of Zhenjiang, Jiangsu Province, China, and were transported to the laboratory in clean paper bags. Once at the lab, the snails were washed three times with dechlorinated water and placed onto plates for 24 h. The snails that migrated out of the plate and acclimatized to the laboratory environment for 1 week were placed in clean large plates, with paper on the bottom. Each plate contained 1,000 snails, at RT under 12 h light:12h dark photoperiods. Meanwhile, unpolluted soil was accumulated, dried, and crumbled before passing through an 840-μm mesh brass sieve to generate snail feed. 0.50 g of the snail feed was then distributed onto each plate every 7d. Finally, active adult snails, containing 7–8 spirals, were randomly assigned to different groups for the molluscicidal examination. The Institutional Committee for the Care and Use of Laboratory Animals (Jiangsu Institute of Parasitic Diseases) approved the procedures for snail culture, sacrifice, and tissue sampling (JIPD-2020-005).

### Molluscicidal assay

The test compounds were prepared in 100 mL flasks in the following final concentrations: 0.02, 0.03, 0.06, 0.12, 0.25, 0.50, 1.00 and 2.00 mg/L, with <0.01% of Dimethyl sulfoxide (DMSO). In preliminary examinations, only concentrations of 1.00 and 2.00 mg/L were used for each compound. However, for the LC_50_ tests, concentrations of 0.02, 0.03, 0.06, 0.12, 0.25, 0.50, and 1.00 mg/L were used for each compound. To test molluscicidal activity, 10 snails were introduced to 100 mL flasks and the opening was wrapped with gauze to prevent snails from escaping. The snails were kept immersed in corresponding compound solutions for 24, 48 and 72 h at 25 °C, before being washed with dechlorinated water three times, and fed for 48 h at 25 °C. Lastly, they were assessed using the knocking method (Gönnert, 1961; Webbe, 1961). Niclosamide was used as a positive control, whereas DMSO was used as a negative control. The molluscicidal activity test was performed thrice.

### Extraction of ATP and ADP from snail soft tissue

*O. hupensis* snails were treated with C6 at LC_50_ and 1/2 LC_50_ or DMSO (negative control) for 24 h. Following this, the soft tissues (100 mg) of the surviving snails were homogenized with 2 mL of 0.6 mol/L perchloric acid in an ice bath for 1 min. The extraction solution was then centrifuged at 10,000×*g*, 10 min for 4 °C, and 1 mL of the supernatant was quickly neutralized to pH = 6.5–6.8 with 1 mL of KOH solution and allowed to remain in an ice bath for 30 min to allow for the precipitation of potassium perchlorate. After a second centrifugation at 10,000×*g* for 10 min at 4 °C, the potassium perchlorate was removed and the supernatant was filtered through a 0.45 μm filter and stored at RT until further analysis (Bhatt et al., 2012; Menegollo et al., 2019).

### ATP and ADP assessment using HPLC

#### Standard stock solution preparation

ATP and ADP standards (1 mg) were separately re-suspended in 10 mL of deionized water to prepare 100 μg/mL ATР and ADP standard stock solutions. To achieve concentrations of 40, 20, 10, 5, 2.5 and 1.25 μg/mL, the stock solution was diluted accordingly using deionized water. HPLC analysis was performed with five μL of each sample.

#### Mobile phases (MP)

MP A was composed of 3.40 g/L dipotassium hydrogen phosphate and 20 μg/L tetrabutyl ammonium re-suspended in deionized water and adjusted to pH = 6.4 with 0.1 mol/L potassium hydroxide. MP B was 100% methanol. Air bubbles in solutions were eliminated with ultrasonic technology.

#### Chromatographic condition

The HPLC parameters were as listed below: an AgilentZORBAX SB-C18 column was equipped with an Agilent 1260 system. Peak detection and analysis was conducted at 254 nm. MP flow rate was 1.0 mL/min and the injection volume was 20 mL. The sample ATP and ADP were recognized according to the standard retention time. The concentrations of ATP and ADP, on the other hand, were calculated using an external standard method.

#### Recovery trial

The ATP and ADP standards (0.1 mL, 400 μg/mL) were added to the snail soft tissue. and the extraction was carried out, as described above. The experiment was repeated six times.

### Mitochondrial respiratory chain complex activity

DMSO solution containing C6 was added to the reaction system to prepare the final concentrations of 1/2LC_50_ and LC_50_ (24 h). The activity of the mitochondrial respiratory chain complex was measured according to the procedure of activities mentioned in the mitochondrial respiratory chain complex I, III, and IV test kits (Solarbio Beijing, Beijing, China), and DMSO was used as a blank control. One-way analysis of variance (ANOVA) was used to analyze the complex activity data with the help of the SPSS17.0 software.

### Mitochondrial membrane potential

Mitochondria were extracted using the Mitochondria isolation kit (Solarbio Beijing, Beijing, China). The 1/2LC_50_ and LC_50_ (24 h) of C6 were achieved after adding the DMSO solution containing C6 to the reaction system. Carbonyl cyanide 3-chlorophenylhydrazone (CCCP) was used as the positive control. The mitochondrial membrane potential was measured according to the procedure indicated on the mitochondrial membrane potential kit (Solarbio Beijing, Beijing, China), and DMSO was set as the blank control.

## Results

We tested 10 ADs for corresponding molluscicidal activity against adult *O. hupensis*. The molluscicidal data spanning 24, 48, and 72 h are presented in Table 1. Based on our results, six compounds, namely C1, C2, C3, C6, C7, and C8 exhibited strong toxicity toward *O. hupensis*, at a concentration of 1 mg/L. However, at the same concentration, C4, C5, C9, and C10 exhibited <50% lethality % at 24, 48, and 72 h. We have, therefore, selected the former compounds (*i.e*., C1, C2, C3, C6, C7, and C8) for further experimentation. To assess the toxicity of these compounds on *O. hupensis*, we evaluated LC_50_ values at varying exposure times, using bioassay data probit analysis (Table 2). C2 displayed the most toxicity at 24 and 48 h, whereas C6 was most effective at 72 h. C2 was highly toxic to snails at a concentration of ≥0.25 mg/L at 24 and 48 h, and was not toxic at concentrations of ≤0.02 mg/L. Additionally, the LC_50_ of C2 at 24, 48, and 72 h were 0.23, 0.14, and 0.14 mg/L, respectively, and the LC_50_ of C6 at 24, 48, and 72 h were 0.27, 0.19, and 0.13 mg/L, respectively. Interestingly, the elimination of Br from the pyrrole ring of C3 diminished molluscicidal activity from 100% (C2) to 20–40% (C1) at the concentration of 0.25 mg/L. Likewise, the toxicity of C4 and C9, as compared to C3, declined at varying degrees. These data suggest an essential role of the Br on pyrrole in inducing *O. hupensis* mortality. Additionally, altering the substituent (1) on the pyrrole ring produced varying levels of toxicity. Of note, the molluscicidal activities of C3, C5, C6, C7, C8, and C9 were similar to C2, but the largest snail mortality achieved with C10 was only 50% at 1 mg/L.

**Table 1:** Preliminary molluscicidal activity of ADs against *O. hupensis* snails at 1 mg/L.

Compound	Exposure time (h)	Mortality (%) (*n* = 30)
1	24	50.00
	48	93.33
	72	96.67
2	24	100.00
	48	100.00
	72	100.00
3	24	40.00
	48	90.00
	72	100.00
4	24	20.00
	48	33.33
	72	46.67
5	24	20.00
	48	20.00
	72	50.00
6	24	100.00
	48	96.67
	72	100.00
7	24	33.33
	48	100.00
	72	100.00
8	24	60.00
	48	100.00
	72	100.00
9	24	20.00
	48	20.00
	72	20.00
10	24	43.33
	48	46.67
	72	43.33
Niclosamide	24	100.00
	48	100.00
	72	100.00
Control	24	0
	48	0
	72	3.33

**Table 2:** Molluscicidal activity of AD against *O. hupensis* snails.

Compound	Time (h)	Mortality rate of snails in different concentrations (%) (*n* = 30)							LC50 (mg/L)
		1.00 mg/L	0.5 mg/L	0.25 mg/L	0.12 mg/L	0.06 mg/L	0.03 mg/L	0.02 mg/L	
1	24	40.00	36.67	20.00	6.67	6.67	0.00	0.00	–
	48	93.33	73.33	20.00	13.33	16.67	0.00	0.00	0.37
	72	100.00	60.00	43.33	10.00	3.33	0.00	0.00	0.33
2	24	96.67	90.00	50.00	20.00	20.00	3.33	0.00	0.23
	48	100.00	100.00	100.00	33.33	10.00	3.33	0.00	0.14
	72	100.00	100.00	100.00	30.00	6.67	0.00	0.00	0.14
3	24	40.00	16.67	10.00	10.00	0.00	0.00	0.00	–
	48	80.00	60.00	70.00	40.00	10.00	10.00	0.00	0.22
	72	100.00	80.00	30.00	30.00	30.00	10.00	0.00	0.25
6	24	100.00	90.00	30.00	30.00	10.00	0.00	0.00	0.27
	48	96.67	86.67	60.00	30.00	20.00	10.00	0.00	0.19
	72	100.00	96.67	100.00	30.00	0.00	10.00	0.00	0.13
7	24	33.33	40.00	20.00	10.00	0.00	10.00	0.00	1.68
	48	100.00	56.67	50.00	40.00	3.33	10.00	0.00	0.25
	72	100.00	100.00	63.33	70.00	20.00	10.00	0.00	0.11
8	24	60.00	50.00	20.00	23.33	30.00	6.67	0.00	0.64
	48	100.00	90.00	20.00	20.00	30.00	10.00	0.00	0.33
	72	100.00	80.00	80.00	30.00	10.00	20.00	10.00	0.16
Niclosamide	24	100.00	80.00	40.00	26.67	0.00	0.00	0.00	0.14
	48	100.00	100.00	90.00	90.00	46.67	20.00	0.00	0.03
	72	100.00	100.00	100.00	100.00	63.33	10.00	0.00	0.03
Control	24	0	–	–	–	–	–	–	–
	48	0	–	–	–	–	–	–	–
	72	3.3	–	–	–	–	–	–	–

**Figure 2:**
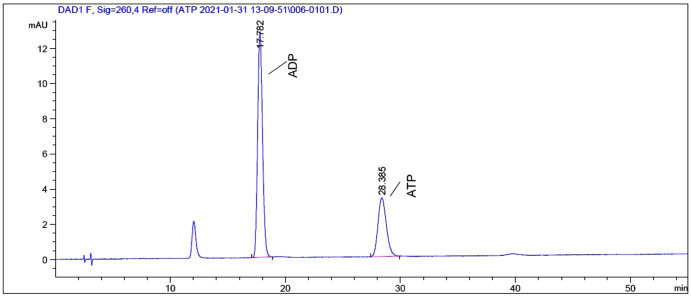
HPLC chromatogram of a standard mixture of ATP and ADP.

**Figure 3:**
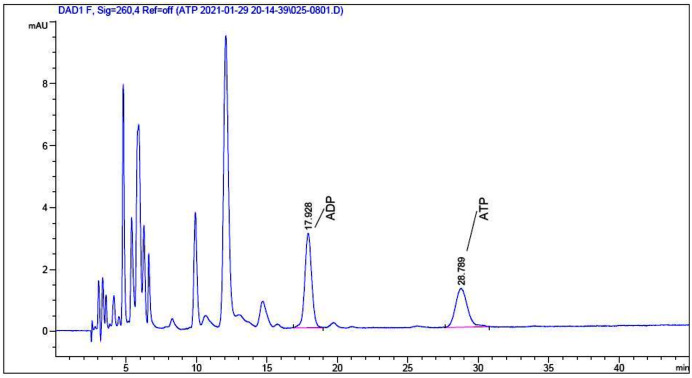
HPLC chromatogram of ATP and ADP extracted from *O. hupensis* snail soft tissue.

**Figure 4:**
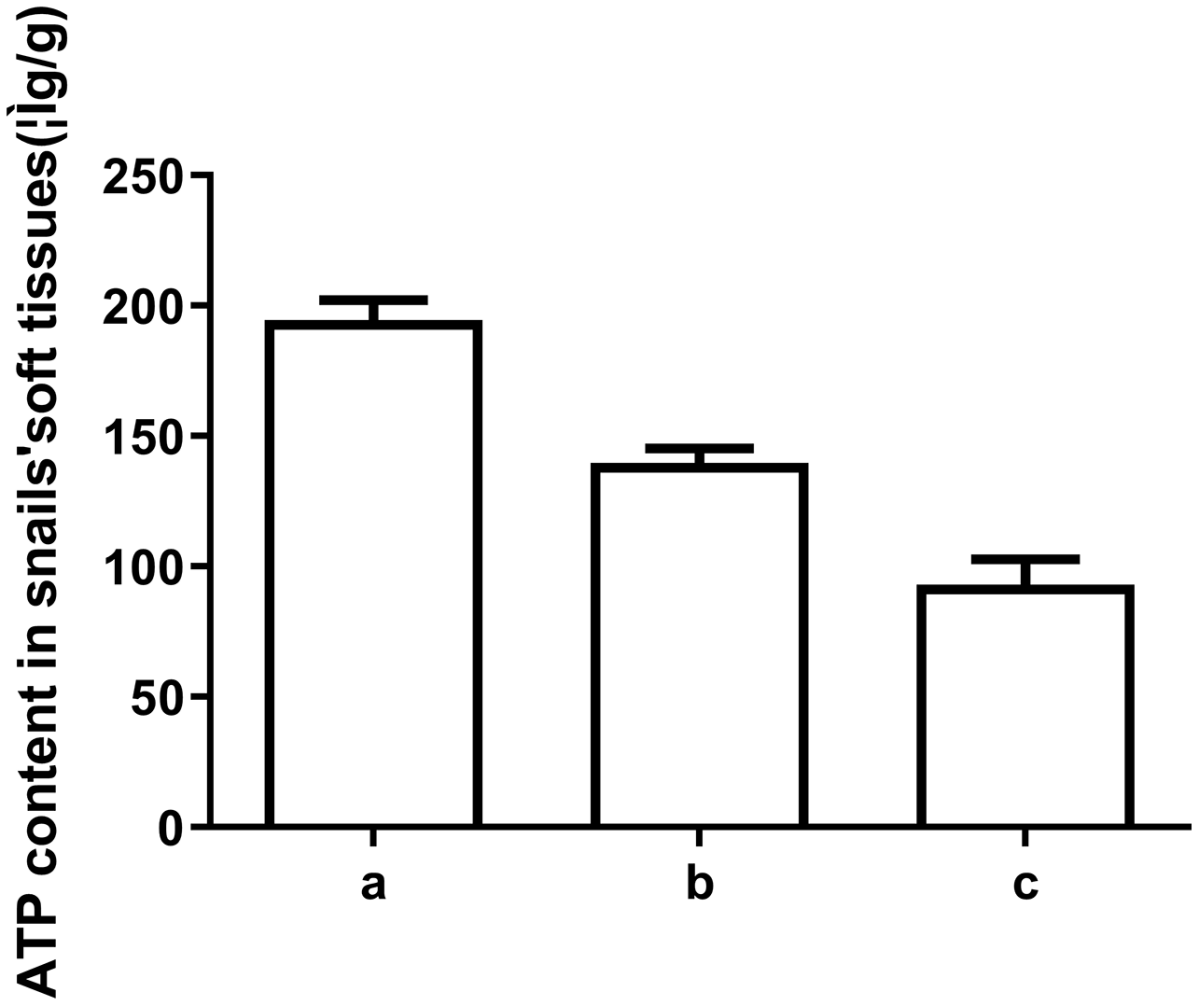
ATP levels in snail soft tissues after exposure to LC50 and 1/2 LC50 of compound 6. (A) Control. (B) Treated with 1/2 LC50 (C) Treated with LC50 (*n* = 3).

**Figure 5:**
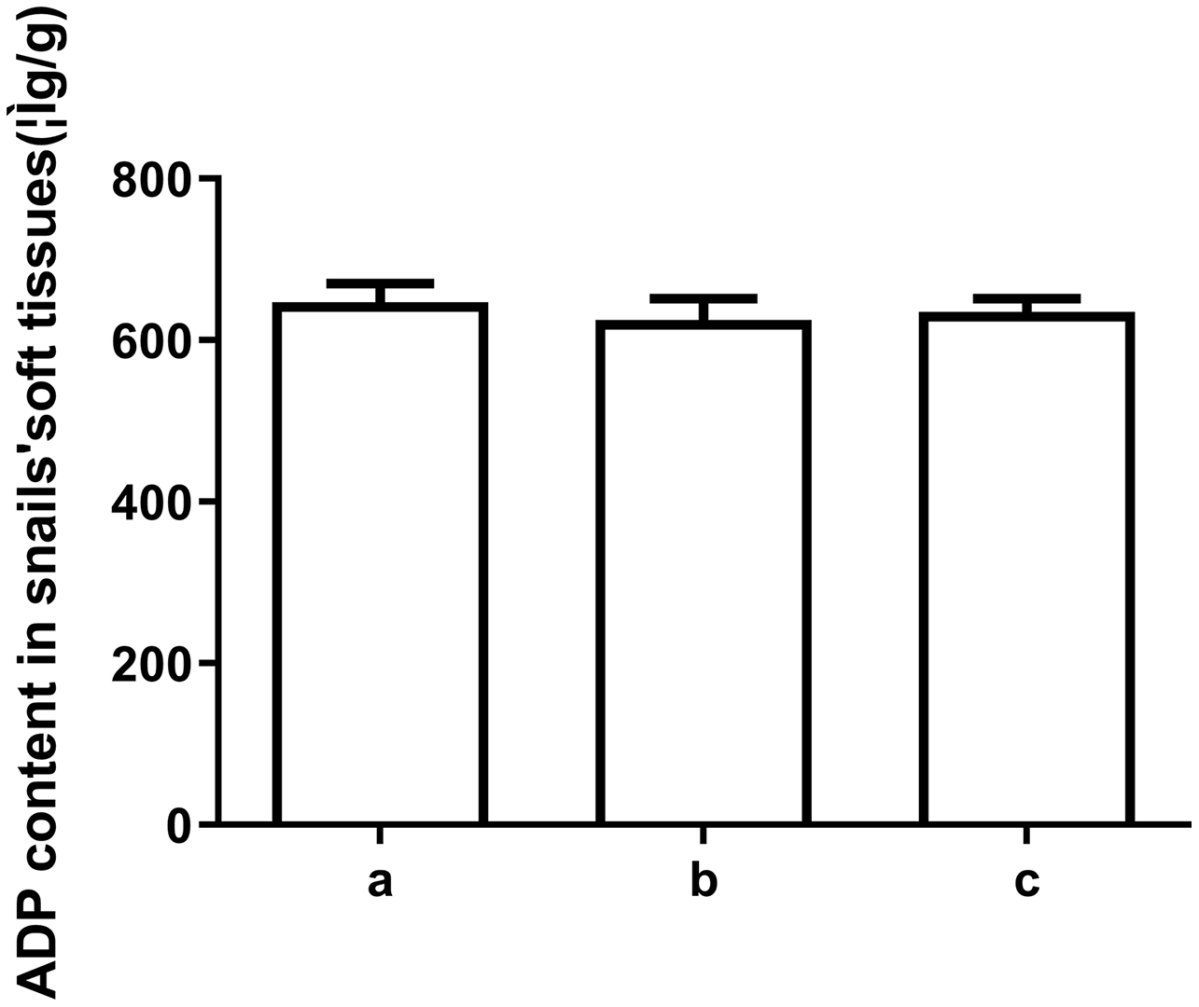
The ADP levels in snail soft tissues after exposure to LC50 and 1/2 LC50 of compound 6. (A) Control. (B) Treated with LC50 (C) Treated with 1/2 LC50 (*n* = 3).

To explore the underlying mechanism behind the toxicity of ADs on snails, we assessed ATP and ADP levels within the soft tissues of snails after exposure to C6, using HPLC. As depicted in Figs. 2 and 3, ATP and ADP were successfully isolated and detected at 254 nm. We discovered a linear relationship among ATP and ADP levels, with a range of 1.25–40 μg/mL against their peaks and a coefficient of determination of 0.9993 and 0.9973, respectively. The mean recovery rates of ATP and ADP were 97.52% and 91.28%, respectively. The CV of ATP and ADP were 4.51% and 2.51%, respectively (Table 3). Next, we measured the ATP and ADP content within the soft tissues of snails after exposure to the toxic compounds. We revealed a marked reduction in ATP levels from 194.46 to 139.75 μg/g after treating snails with half LC_50_ and further reduction to 93.06 μg/g at when exposed to LC_50_ (Fig. 4). On the contrary, ADP levels remained the same both before and after toxic compound exposure (Fig. 5).

**Table 3:** Comparative and recovery rates of HPLC analyses of ATP and ADP.

Item	Mean peak areas (mv*min, *n* = 6)	Mean recovery rate (%)	Coefficient of variation (%)
ATP	370.2	97.52	4.51
ADP	430.8	91.28	2.51

**Figure 6:**
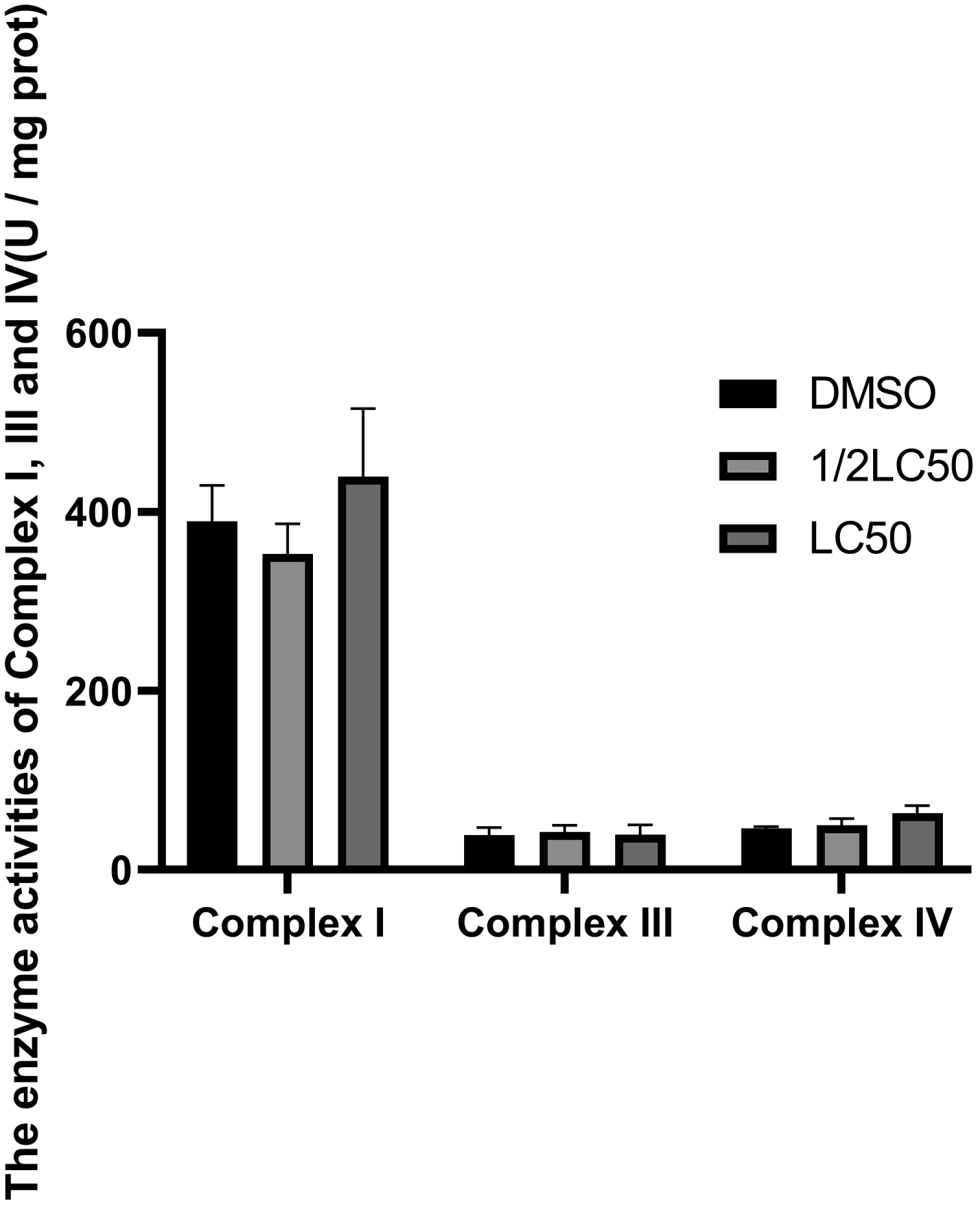
Alterations in enzymal activities of Complex I, III, and IV of *O. hupensis* snails exposed to C6.

**Figure 7:**
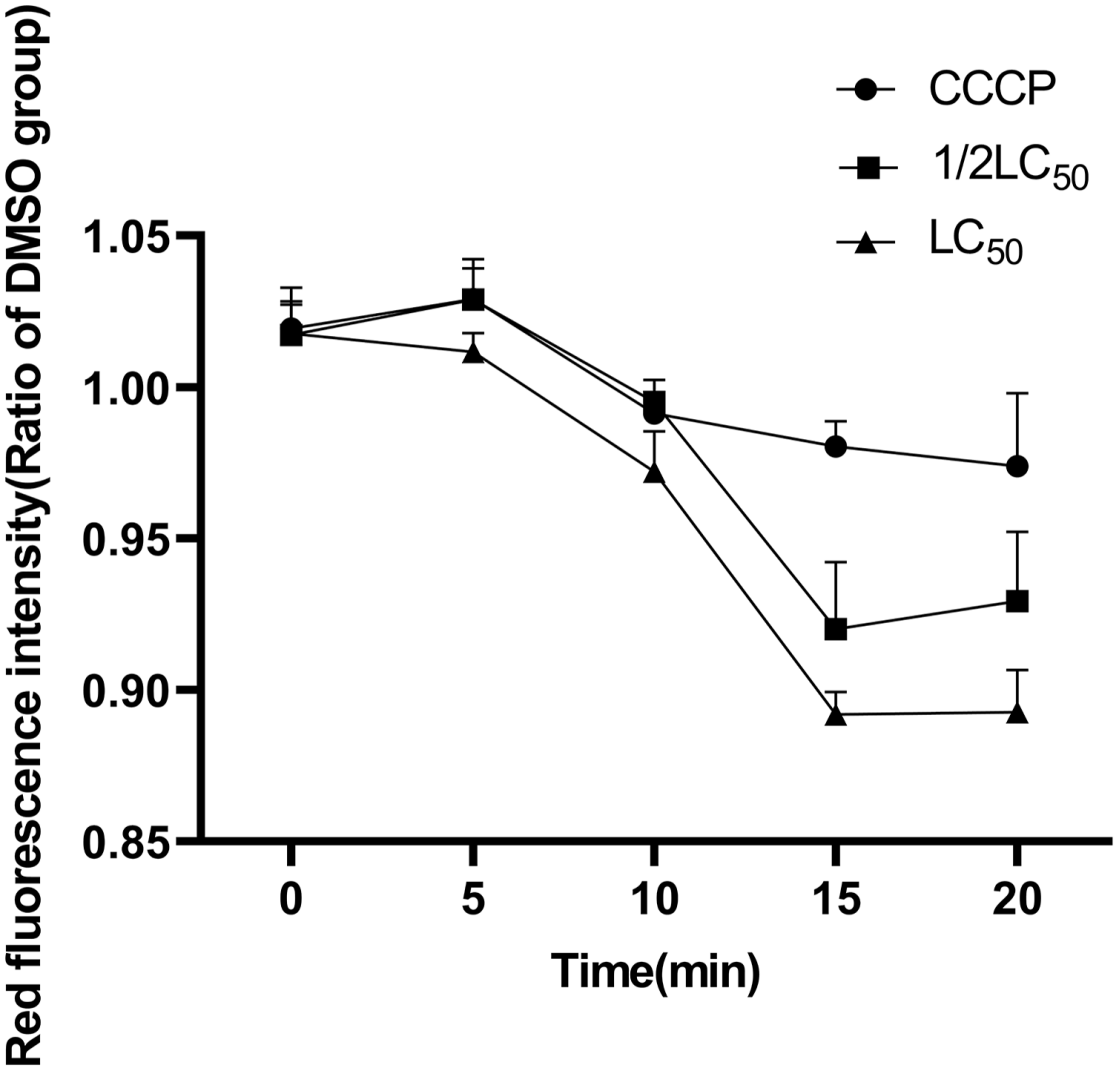
Effects of C6 on mitochondrial membrane potential of *O. hupensis* snails. The concentration of CCCP was 0.10 mg/L (*n* = 3).

The mitochondrial respiratory chain complex I, III, IV and the mitochondrial membrane potential are key points of energy metabolism. The activities of enzymes and the mitochondrial membrane potential are often measured to evaluate the effects of ATP reduction and determine possible mechanisms. As shown in Figs. 6 and 7, 1/2LC_50_ were enough to alter the biochemical parameters of the snail. The mitochondrial membrane potential decreased after exposure to C6 when compared with the control group. No obvious change in the activities of the mitochondrial respiratory chain complex I, III and IV was observed.

## Discussion

The *Oncomelania* snail presents along and south of the Yangtze River and acts as an intermediate host to the parasite *S. japonicum*. Given the detrimental effects of the *S. japonicum* in humans, special effort is underway to eliminate the intermediary host, *i.e.*, the *O. hupensis* snail (Mccullough et al., 1980; Moné, 2017). As such, molluscicides have become common due to its rapid effect and ease of use. The only molluscicide recommended by WHO is Niclosamide (Dai et al., 2008). Although Niclosamide has relatively low toxicity toward humans and domesticated animals and exerts no detrimental effect on crops, its high cost and toxicity toward aquatic animals is of major concern (Andrews, Thyssen & Lorke, 1982). At present, snail control is facing a new challenge due to the implementation of the national strategy of the Yangtze River Economic Belt (Jiani, 2017; Xu, Wu & Zhang, 2020) that ensures environmental protection. Therefore, Niclosamide can no longer be used due to its toxicity towards aquatic animals and the need to develop novel molluscicides, that would kill snails without harming the surrounding ecosystem, has become both necessary and urgent.

In this study, we synthesized 10 ADs carrying various structural modifications on the pyrrolyl group. Using activity analysis, we showed that altering the substituents on the pyrrolyl group can vastly change the potency. Thus, by removal of bromide from the pyrrole ring, the snail mortality of C3, at a concentration of 0.25 mg/L, was reduced from 100% (C2) to 20–40% (C1). Likewise, the activities of C4 and C9, as compared to C3, declined by varying degrees. We also found that the bromide substitution on the pyrrole ring was essential for molluscicidal activity. As such, C2 exhibited the strongest activity within 24 and 48 h of exposure, whereas C6 was most active at 72 h, suggesting that C6 had a slower rise in potency than C2. Various results suggested C2 to be the most effective molluscicide. In fact, compounds that are substituted with pyrrole 1 are generally products of C2, which can be converted back to C2 by enzymes in target organisms (Black et al., 1994). In our study, we demonstrated that the activities of C3, C5, C6, C7, and C8 remained the same as C2, which may be due to the conversion of these compounds back to C2 within *O. hupensis*. Moreover, their molluscicidal effect appeared slowly, indicating that these compounds may be precursors to C2 and should be investigated in detail. Interestingly, according to LC_50_, the activities of C2 and C6 was superior to others. Given that C2 is detrimental to plants, our investigation focused on C6. Noteworthily, C10 had relatively low activity, which may be due to an inability to be converted to C2 inside *O. hupensis*.

A few mechanisms of action have been highlighted by studies on molluscicides and snail mortality. In the current study, multiple molluscicidal drugs were shown to diminish energy metabolism *via* downregulation of its enzymes, leading to biological death. ATP is one of the major players of energy metabolism and cell death (Dawes & Senior, 1973; Harrison & Maitra, 1968). It carries chemical energy within cells and fuels the synthesis of proteins and membranes, movement of cell, cellular division, and so on (Bershadsky & Gelfand, 1983). We have, therefore, evaluated the levels of ATP and ADP in *O.hupensis* soft tissue after exposure to the synthesized compounds, using HPLC. We found that, out of the 10, 6 compounds diminished ATP levels, at sublethal concentrations, and produced snail death. We also found that the reduced ATP production was positively correlated with the concentration of the C6. The mitochondrial respiratory chain complex I, III, IV, and the mitochondrial membrane potential are key points of ATP production. It is clear from the results that C6, at sub-lethal concentrations, can disturb the mitochondrial membrane potential, and disrupt snail ATP production, resulting in death.

## Conclusions

Here, a number of ADs were designed and synthesized. The substituents on the pyrrole ring were sequentially modified and the corresponding molluscicidal potency was tested against *O. hupensis*. We demonstrated that the bromide substitution on the pyrrole ring is essential for molluscicidal activity. Among the compounds studied, C6 showed strong molluscicidal property, based on its ability to disturb the mitochondrial membrane potential which is necessary for ATP production, To explore the development potential for use as a novel molluscicidal agent, further investigations are in progress. Thus, field trials, toxicity studies towards non-target aquatic organisms, will be reported soon.

## Supplemental Information

10.7717/peerj.12209/supp-1 Supplemental Information 1 ATP and ADP in *O. hupensis* soft tissue after exposure to the synthesized toxic compounds by high performance liquid chromatography. All content unit is μg/g, All concentration unit is μg/mL

10.7717/peerj.12209/supp-2 Supplemental Information 2 Alterations in enzymal activities of Complex I, III, and IV of *O. hupensis* snails exposed to C6.

10.7717/peerj.12209/supp-3 Supplemental Information 3 Effects of C6 on mitochondrial membrane potential of *O. hupensis* snails.

10.7717/peerj.12209/supp-4 Supplemental Information 4 Compound NMR spectra.

## Additional Information and Declarations

### Data Availability

The following information was supplied regarding data availability: The raw measurements are available in the Supplemental Files.
