# Supplementary material for: Evaluation of the molluscicidal activities of arylpyrrole on Oncomelania hupensis, the intermediate host of Schistosoma japonicum
Source: PeerJ. 2021 Sep 27;9:e12209. doi: 10.7717/peerj.12209 (PMC8483005; doi:10.7717/peerj.12209)
Supplement: Supplemental Information 4 [file peerj-09-12209-s004.docx]

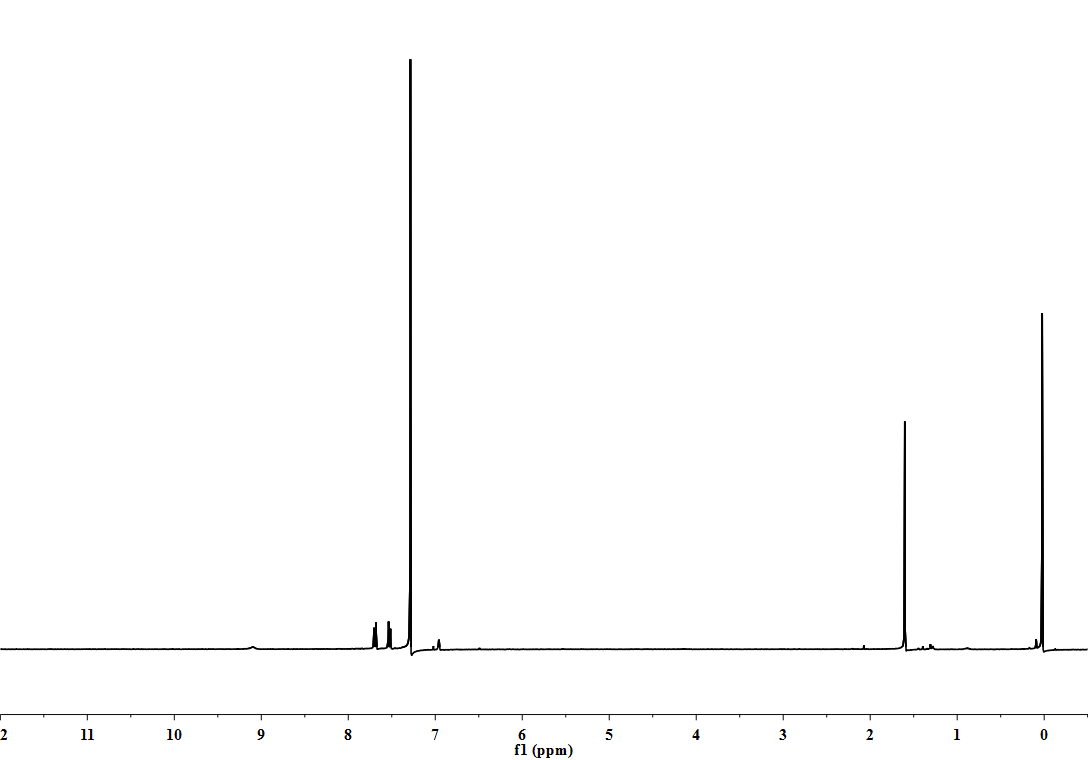


NMR spectra of C1


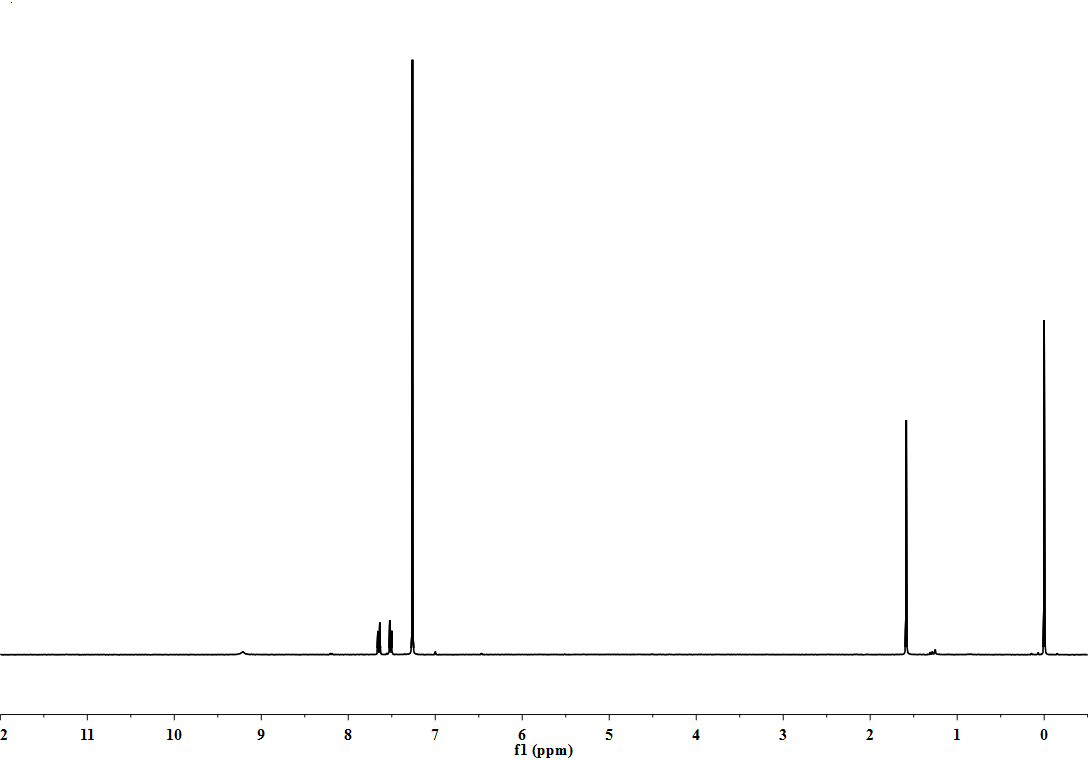


NMR spectra of C2
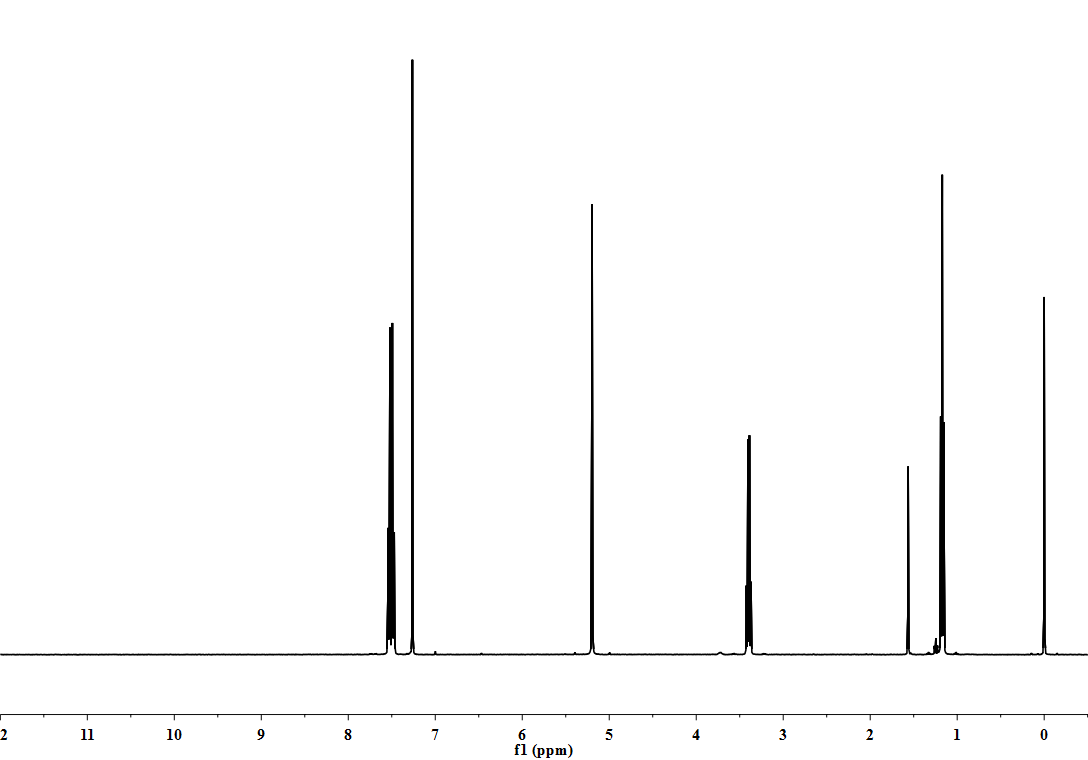


NMR spectra of C3
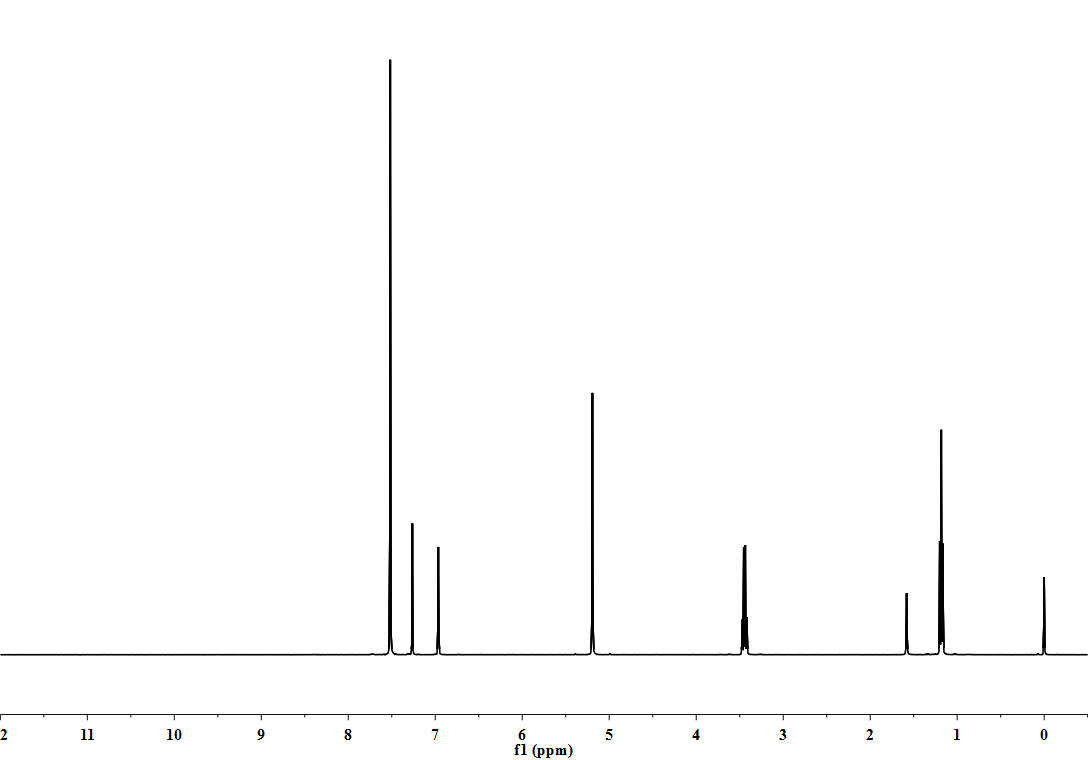


NMR spectra of C4
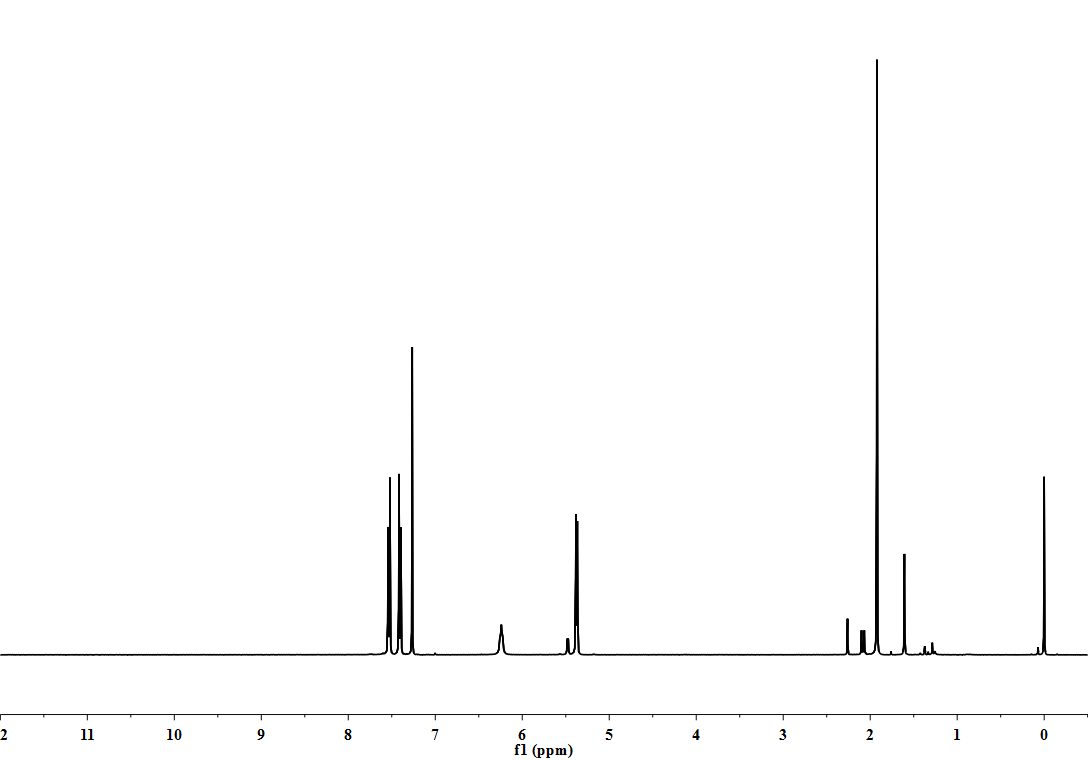


NMR spectra of C5
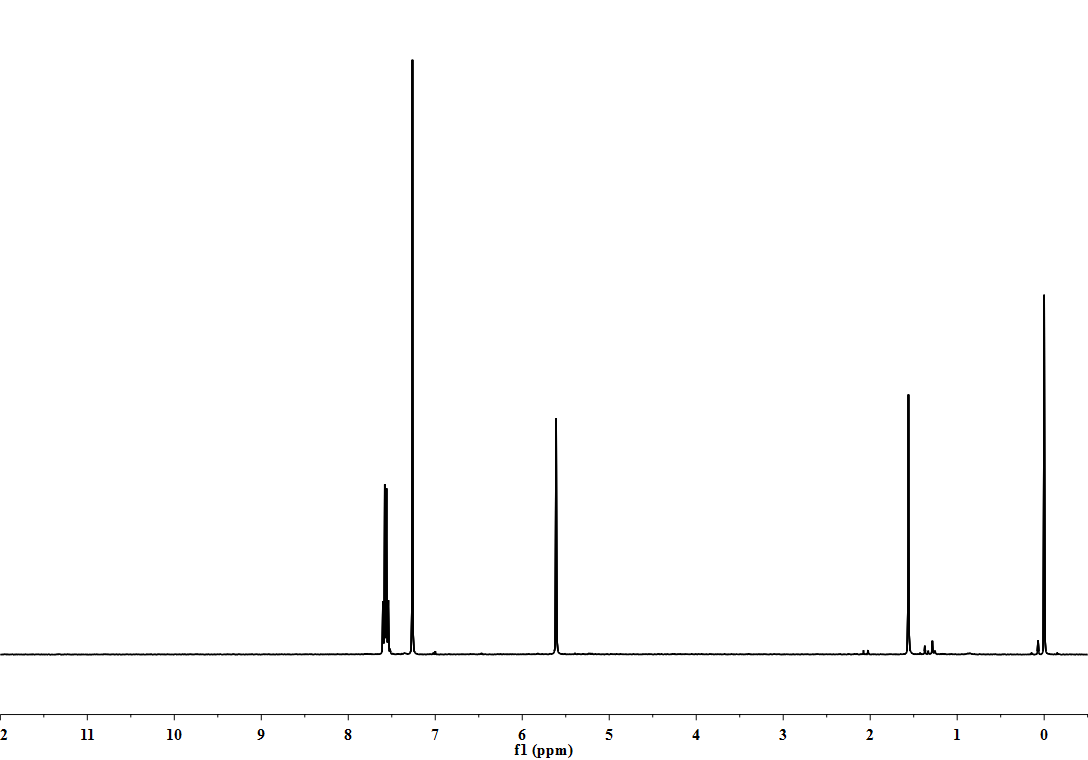


NMR spectra of C6
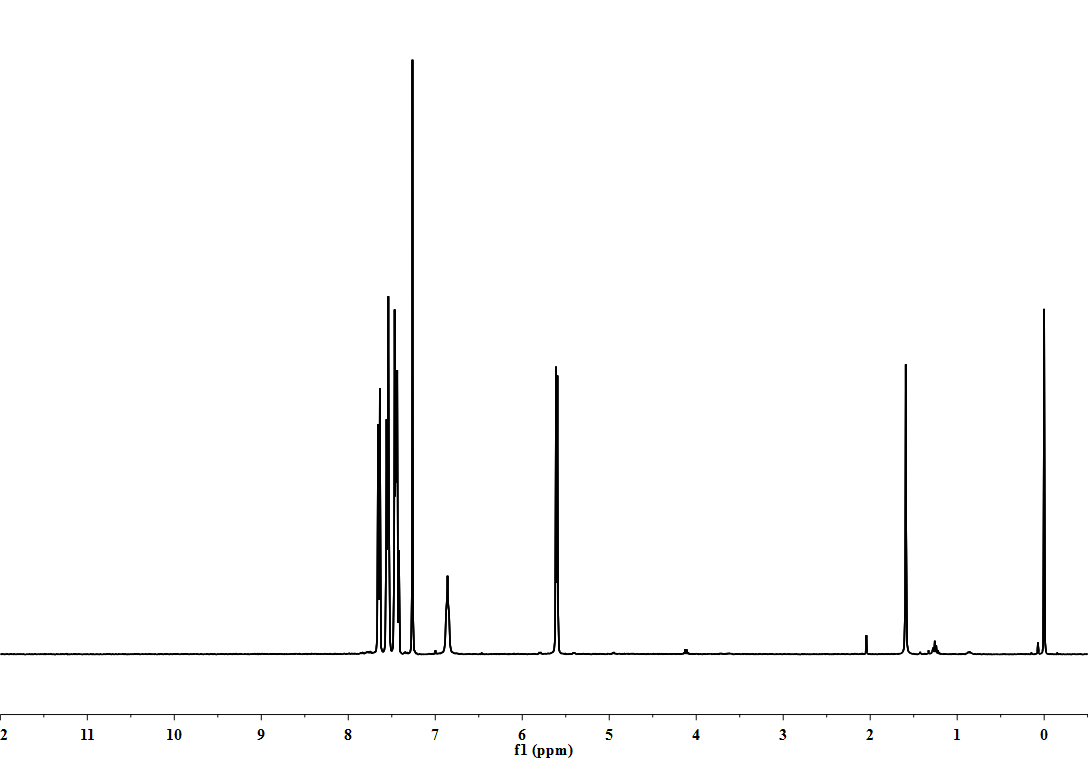


NMR spectra of C7
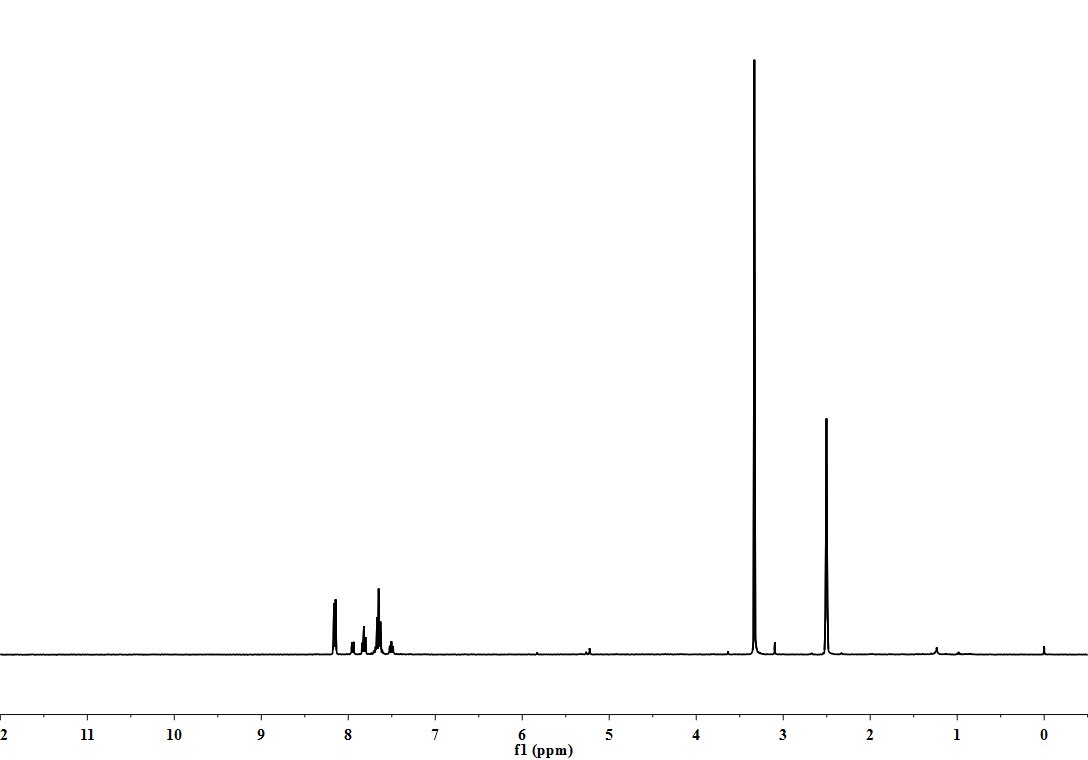


NMR spectra of C8
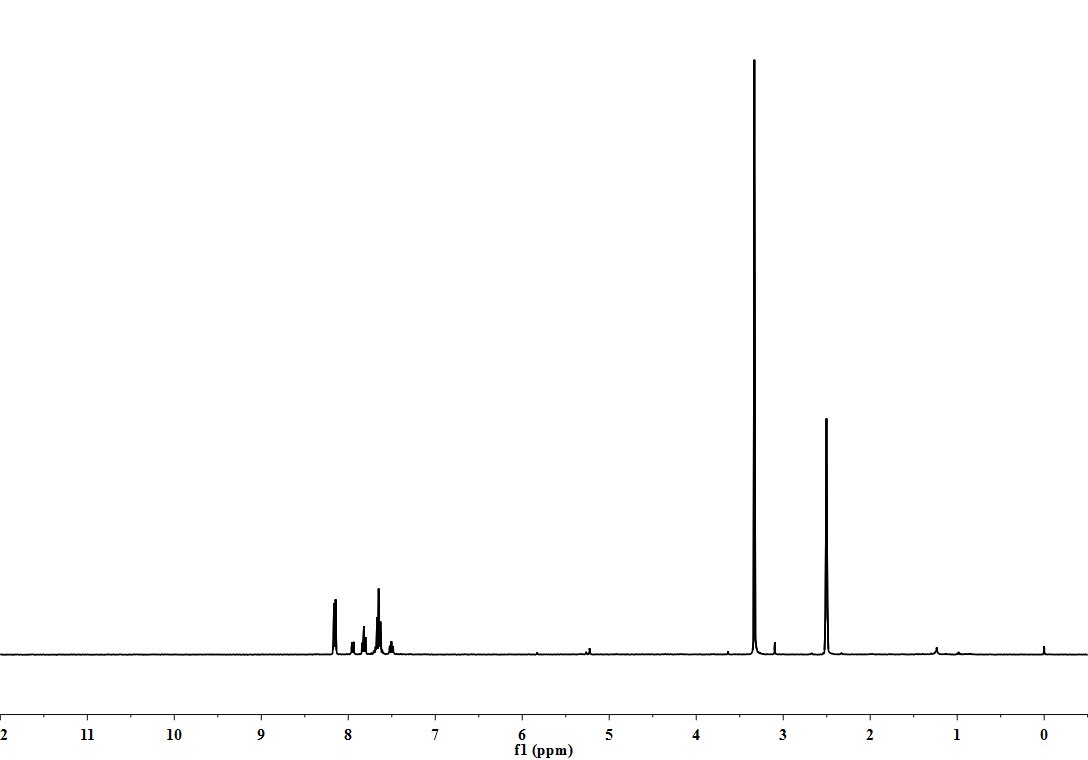


NMR spectra of C9
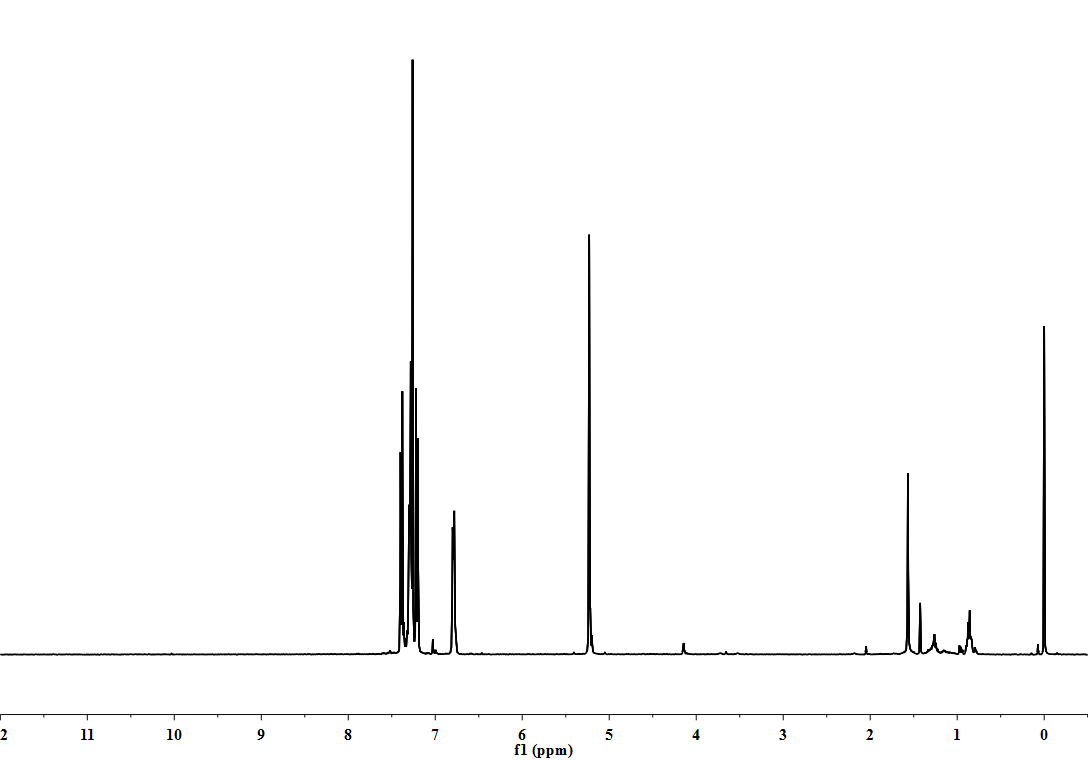


NMR spectra of C10
